# Ossifying tendinitis of the rotator cuff after arthroscopic excision of calcium deposits: report of two cases and literature review

**DOI:** 10.1007/s10195-014-0309-8

**Published:** 2014-07-15

**Authors:** Giovanni Merolla, Arpit C. Dave, Paolo Paladini, Fabrizio Campi, Giuseppe Porcellini

**Affiliations:** 1 Unit of Shoulder and Elbow Surgery, “D. Cervesi” Hospital, AUSL della Romagna, Ambito Territoriale di Rimini, L.V Beethowen 5, 47841 Cattolica, RN Italy; 2 Biomechanics Laboratory “Marco Simoncelli”, D. Cervesi Hospital, Cattolica, AUSL della Romagna,Ambito Territoriale di Rimini, Cattolica, Italy

**Keywords:** Ossifying, Calcifying, Tendinitis, Shoulder, Arthroscopy

## Abstract

Ossifying tendinitis (OT) is a type of heterotopic ossification, characterized by deposition of hydroxyapatite crystals in a histologic pattern of mature lamellar bone. It is usually associated with surgical intervention or trauma and is more commonly seen in Achilles or distal biceps tendons, and also in the gluteus maximus tendon. To our knowledge, there is no description of OT as a complication of calcifying tendinitis of the rotator cuff. In this report, we describe two cases in which the patients developed an OT of the supraspinatus after arthroscopic removal of calcium deposits. The related literature is reviewed.

## Introduction

Subacromial calcium deposits and calcifications in the tendons of the rotator cuff (RC), with histologic presence of chondrocytes along tenocytes, were identified as a cause of scapulohumeral periarthritis in the early 1900s [1–3]. Later, the term calcifying tendinitis (CT) was coined, denoting an evolutionary process tending towards spontaneous healing [4]. Prevalence of CT was reported to be 2.7 % in asymptomatic individuals and it seems to be more common in females between their fourth and sixth decades, and sedentary workers [5]. Uhthoff and Sarkar [6] noted that CT evolves through a typical cycle in three distinct stages: pre-calcific, calcifying and post-calcific. The pre-calcific stage is characterized by metaplasia of tenocytes into chondrocytes that can be stimulated by multiple factors including hypoxia, microtrauma, disuse and hormonal action. The calcific stage can be divided into three phases: formation, resting and resorption; the process evolves from deposition of amorphous calcium phosphate followed by vascularisation to absorb the calcium deposits. The phase of resorption is associated with significant clinical pain experienced by the patient. The post-calcific stage marks collagenisation of the lesion by fibroblasts, thus ending the cycle of calcifying tendinitis.

Ossifying tendinitis (OT) is a type of heterotopic ossification (HO), characterized by deposition of hydroxyapatite crystals in a histologic pattern of mature lamellar bone [7, 8].

It is usually associated with surgical intervention or trauma [9] and is more commonly seen in the Achilles tendon [10] or following repair of ruptured distal biceps [11]. To our knowledge, there is no description of OT as a complication of calcifying tendinitis of the rotator cuff. In this report, we describe two cases in which patients developed an OT of the supraspinatus after arthroscopic removal of calcium deposits, and we review the literature.

## Case report

### Case 1

In April 2005 the patient came to our outpatient office, complaining of severe pain and discomfort in the right shoulder for 1 year. After radiological and ultrasound (US) examination, he was diagnosed with calcific tendinitis of the rotator cuff and he underwent two cycles of extracorporeal shock wave (ESW) therapy. At 1 year follow-up he had not had any improvement in pain and shoulder function and therefore was advised to undergo shoulder arthroscopy.

**Fig. 1:**
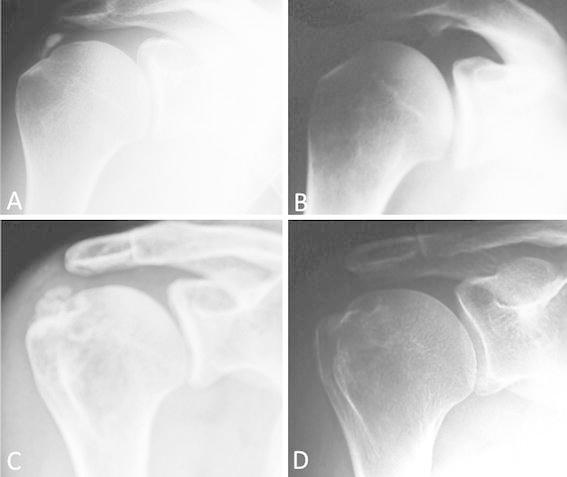
Radiographic evaluation of case 1: **a** preoperative X-ray (November 2005) of the right shoulder showing a subacromial (S/A) calcification of 1.2 cm; **b** postoperative radiographs confirmed the complete excision of the calcium deposit; **c** preoperative X-ray performed before the 2nd operation (February 2011) highlighted recurrence of calcification as a dense area more than 1 cm in size in the S/A space; **d** last follow-up radiographic evaluation confirmed the absence of ossifying mass

Preoperative shoulder examination showed the following range of motion (ROM): 160° in flexion and abduction, 80° in internal rotation(IR) and 90° in external rotation(ER). Impingement tests (Hawkins and cross-arm) and Empty Can Test were found positive. The Constant–Murley score (CS) was 67 points, and the Simple Shoulder Test (SST) had a 5/12 “yes” response. Laboratory exams showed normal values of peripheral blood counts, and X-ray showed a 1.2-cm calcification located on the bursal side of the supraspinatus tendon (Fig. 1a).

**Fig. 2:**
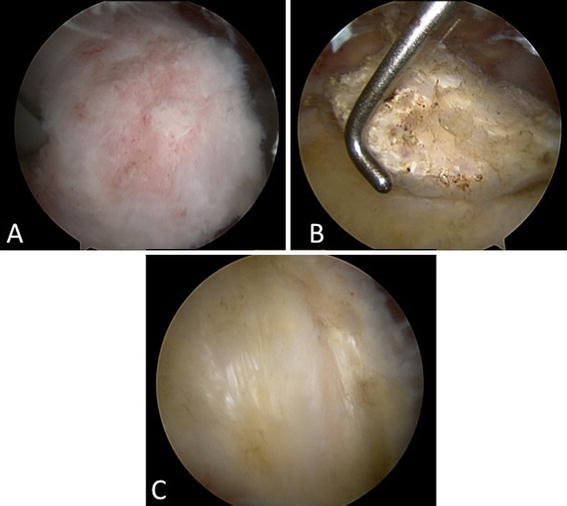
Intraoperative findings of case 1 at the time of the second arthroscopy: **a** a dense and irregular area was found in the S/A space, above the supraspinatus and infraspinatus insertion; **b** the ossifying mass was isolated and prepared to be excised; **c** after the excision of the mass the tendon was found to be intact

**Fig. 3:**
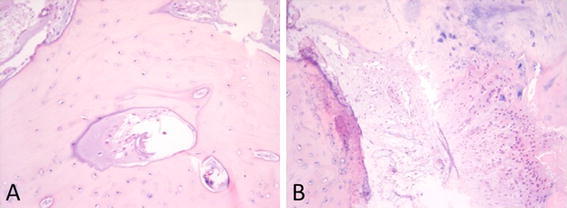
Histologic examination of the mass excised (hematoxylin–eosin ×100): **a** case 1: tendinous tissue with areas of bone metaplasia, **b** case 2: bone and chondroid areas of metaplasia inside the tendinous tissues

The patient underwent shoulder arthroscopy in November 2005. Intraoperatively we found a severe subacromial bursitis and a calcific deposit of 2 cm in the supraspinatus and in the superior portion of the infraspinatus. We performed a complete debridement up to the tendon edges devoid of calcific residuals and multiple needling in the surrounding tissue to ensure complete removal of the calcific deposit. The gap thus created was sutured with two side-to-side stitches and postoperative radiograms confirmed the complete excision of the calcific deposits (Fig. 1b). Postoperative rehabilitation included arm protection in a sling for 15 days, passive mobilization in the scapular plane after 15 days, active exercises from the 5th week and strengthening exercises after 2 months. The patient was followed up in our unit at 45 days, 3, and 6 months, showing a satisfactory shoulder function, till November 2009 when he complained of pain in the right shoulder for 3 months. At this time he was prescribed a standard program of physiotherapy and stretching. X-ray showed a small focus of calcium deposit in the S/A space close enough to the greater tuberosity. We prescribed a cycle of ESW, but due to persistently increasing pain, he was advised for a second arthroscopic look. Before the second operation the pain was severe and the passive ROM was 45° in ER, 45° in IR, 140° in forward elevation (FE); the CS was 71 points and the SST had 7/12 “yes” responses. Laboratory investigations were normal. Preoperative X-ray performed on February 2011 confirmed the presence of a dense 1.5-cm area in the subacromial space that was suspected to be a recurrence of calcium deposit. The patient was operated upon in February 2011 (Fig. 1c) and, intraoperatively, dense hard calcific deposits were found over and into the substance of both supraspinatus and infraspinatus tendons (Fig. 2a–c). However, completed excision of the whole mass did not hamper the integrity of the tendons and so no cuff repair was planned. On histologic examination, the excised mass showed a widespread chondral and bony metaplasia with myxoid degenerative areas (Fig. 3a). Postoperative X-ray confirmed the excision of the mass. At 8 years follow-up examination he was satisfied and pain free; the SST had 12/12 “yes” responses, the CS was 91 points and he had returned to previous work and sport activities. The supraspinatus tendon was anatomically intact and radiological examination confirmed the absence of any calcific or ossified mass (Fig. 1d).

### Case 2

**Fig. 4:**
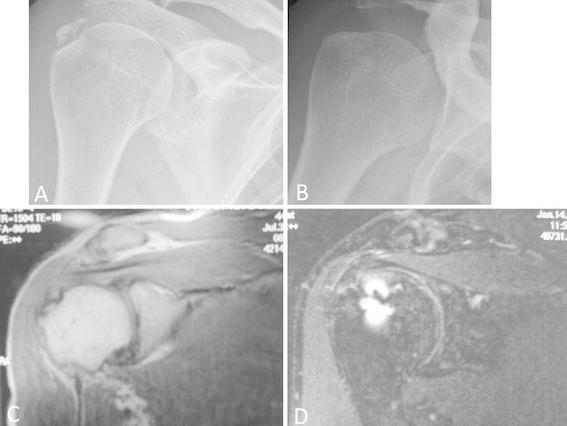
Radiographic evaluation of case 2: **a** preoperative X-ray (April 2006) of the right shoulder with a dense S/A calcification sized 1.5 cm; **b** postoperative radiographs showed the complete excision of the calcific deposit; **c** preoperative MRI (September 2007) before the second operation showed an area of high intensity (T1-weighted) at the supraspinatus insertion that was interpreted as recurrence of calcifying tendinitis with partial tendon tear; **d** MRI before the third operation showed high intensity in the area where the supraspinatus was fixed, but the tendon appeared healed

**Fig. 5:**
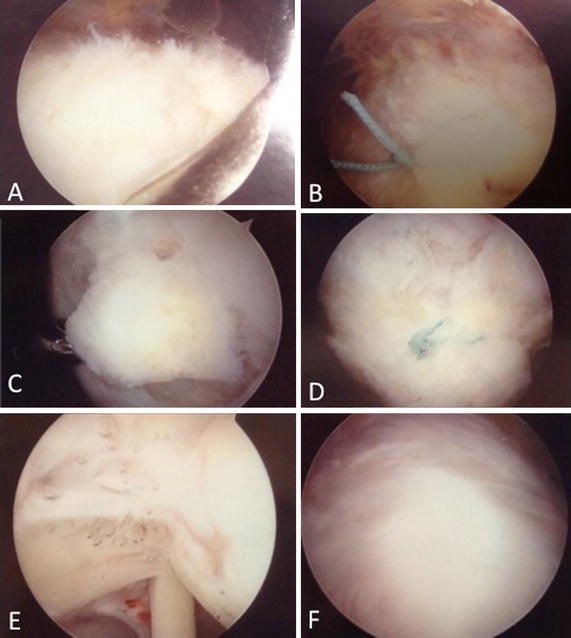
Intraoperative findings in case 2: **a, b** first arthroscopy: the calcium deposit was identified, removed with a motorized shaver and the supraspinatus was repaired with 1 side-to-side suture; **c, d** an ossifying mass was isolated and removed, the tendon tear was repaired with a suture anchor (Super-Revo HI-Fi, ConMed, Largo, Fl, USA); the supraspinatus assessed from the articular (**e**) and bursal side (**f**) appeared completely healed

The patient was evaluated in our outpatient office in April 2005 for severe right shoulder pain and limitation in daily living activity. X-ray and US showed a calcific deposit of 1.5 cm in the supraspinatus tendon. He was given US-guided needling and bursal lavage, but there was no significant pain improvement. No signs of resorption were seen on the X-ray; therefore, in November 2005 he underwent shoulder arthroscopy. Preoperative ROM was 160° in FE, 70° in ER and 80° in IR. CS was 62 points and SST had 3 “yes” responses. Laboratory exams were normal for blood counts and preoperative X-ray confirmed subacromial calcification of the supraspinatus (Fig. 4a). In April 2006, he underwent arthroscopic removal of calcium deposits and 1 side-to-side suture of the supraspinatus tendon (Fig. 5a, b). Complete disappearance of calcification was noted on viewing the postoperative X-ray (Fig. 4b). A standard postoperative rehabilitation was prescribed as for case 1. At the follow-up examinations of 45 days, 3 and 6 months, 1 and 2 years he had a persistent pain with complete range of motion and good supraspinatus strength. In February 2007 he asked to be assessed and we found shoulder and cervical pain with loss of strength in abduction; at this time point US showed lamellar calcification in the subacromial space and he was prescribed shoulder X-rays, and cervical spine MRI. An addition, clinical evaluation by an expert rheumatologist excluded chronic inflammatory diseases and suggested electromyography investigations of the upper limbs that did not reveal alteration in the examined muscle groups. Cervical spine MRI showed no spinal nerve compression nor vertebral body discopathies. He was also assessed for parathyroid hormone, autoantibodies (ANA, ENA, anti ds-DNA, IMF), C-reactive protein (CRP) and eritrosedimentation rate (ASDAS-ESR) but all these laboratory exams were normal. X-rays showed recurrence of calcifications and therefore he underwent MRI that showed high signal intensity of the supraspinatus at its insertion which was considered to be a recurrence of CT with secondary tendon rupture (Fig. 4c). In October 2007 he had a second shoulder arthroscopy where we found a calcific deposit inside the supraspinatus that, when it was grasped with a forceps, appeared to have a consistency similar to bone tissue; the deposit was removed and the tendon was repaired with 1 anchor (Super-Revo HI-Fi, ConMed, Largo, Fl, USA) (Fig. 5c, d). Histologic examination showed chondral and myxoid tissues associated with bony metaplasia foci as found in case 1 (Fig. 3b).

After 1 year of moderate postoperative pain, the patient asked to be reassessed again due to severe disability during work and daily living activities Several attempts at conservative therapies (rehabilitation, laser therapy, NSAID, steroid injections) failed and he was therefore prescribed shoulder MRI that revealed slight changes in signal intensity (T1 weighted) of the supraspinatus insertion due to degenerative alterations of the tendon (Fig. 4d). A third arthroscopic approach in May 2009 showed a subacromial bursitis with fibrous adhesions and a complete tendon healing. We performed a S/A bursectomy, removal of adhesions and tendon stimulation with low radiofrequency (Fig. 5e, f).

The patient followed the standard postoperative program and he had slight pain for 3 years, especially during work activity. At the last FU examination in December 2013 (8 years) the CS was 87 and SST had 10/12 “yes” responses.

## Discussion

CT of the shoulder is a widespread clinical condition with a significant impact on patient’s quality of life. Although several treatments have been proposed, the best option to choose is still controversial [12–16]. Extracorporeal shock wave therapy (ESWT) has been described to be effective [13, 14], but a long-term follow-up study showed that about 20 % of the patients treated have required surgery [14]. US-guided needling, irrigation and aspiration may reduce pain and stimulate calcium resorption [15], while a surgical approach is suggested in cases with persistent disabling symptoms for at least 6 months [16]. Some case-series studies reported good results with partial removal of the calcific deposits, so as to preserve the integrity of the tendon [17, 18]. However, in cases with arthroscopic removal of large and deep calcific deposits, it is recommended to repair the defect with side-to-side sutures or anchors [16, 18]. Recurrence is a known complication following surgical excision of calcific deposits of the shoulder with an incidence reported between 16 % and 18 % [19], but to our knowledge, there is no description of recurrence in the form of OT. Tendon involvement by HO was found in 26.7 % of patients after shoulder surgery and 80 % of these occurred after RC repair and acromioplasty, but the presence of ossifications seemed to be of minor clinical impact [7, 8, 20, 21].

In a case series of 892 patients treated with acromioplasty and distal clavicle resection, Berg et al. [22] reported 5 % with ectopic bone formation, including sites like S/A space, acromio-clavicular joint, coraco-acromial ligament and coraco-clavicular ligament: around 3.2 % of them were symptomatic.

HO of the deltoid muscle [23] and supraspinatus tendon [21] has also been described following open RC repair. The first was managed with resection of pathologic bone and soft tissue contracture by open interval release and manipulation followed by radiation therapy; in the second case the authors did not perform any additional surgery but they described the association with axillary nerve palsy and they highlighted that there were several risk factors present, including two operations within 2 months, smoking and chronic pulmonary disease. In fact, it has been postulated that hypoxia may drive metaplasia in bone-forming cells in patients who are chronic smokers and continue smoking in the peri-operative period and in patients suffering from chronic pulmonary diseases [22, 23].

The mechanism of origin of bone metaplasia in the RC tendon with calcium deposits is unknown, but some aspects of this phenomenon can be interpreted through the findings already known to us. The presence of resident progenitor cells with multi-differentiation potential in the human tendon [24] and local release of bone morphogenic proteins (BMP) which helps in differentiation of pluripotent mesenchymal cells into osteoblasts [25, 26] has been noted after acromioplasty and in cases with degenerated cuff tissue; these biologic changes may thus induce ectopic bone formation [22, 27]. Ectopic chondrogenesis and ossification have been reported in the patellar calcific tendinopathy rat model and to a lesser extent, in the traumatic patellar tendon injury model [28]. The authors detected BMP-2 protein in the chondrocyte-like cells and calcific deposits in both injury models but not in control samples, indicating that BMP-2 might be involved in the pathogenesis of ectopic chondrogenesis and ossification. HO is common after traumatic injuries requiring prolonged immobilization and rigorous passive physiotherapy [9] or can be associated with other specific rheumatic conditions [29]. An additional predisposing factor for HO is an altered balance within the autonomic nervous system, as seen in brain, spinal cord or peripheral nerve injury [30]. Finally, it can develop after minimally invasive surgery and arthroscopy, but the incidence is less common than after open shoulder surgery [31]. The dilution of osteoinductive marrow elements with irrigation fluid and also its continuous washout may be implicated in its formation [23]. We accurately investigated overall features of both our patients but we didn’t find any of the supposed risk factors which are implicated in HO. Both patients were non-smokers with no history of any chronic neurological or internal diseases, and surgeries were performed arthroscopically without pre- or postoperative nerve involvement. The patients followed a protocol of physiotherapy as standardized for all our cases of CT arthroscopically managed. No significant anthropometric difference was found comparing the two patients, nor did they have a family history of inflammatory osteoarthritis, connectivitis or other rheumatic or metabolic disorders; both were employed with no potential habits (smoking, alcohol, drugs, dietary behaviour) or work-related risk factors.

In both cases the RC was involved with severe pain and functional impairment that required an arthroscopic second look to ascertain the origin and the characteristics of the mass.

During the surgical procedure of case 1 we found a formation of hard consistency above and partly within the supraspinatus tendon at the same site the first calcific deposit was removed from; in case 2 we found similar macroscopic characteristics of the ossification, with a tendency to infiltrate the tendon. The histologic features showed in both cases areas of chondrometaplasia and ossification that were diagnosed as a particular form of OT, without supposing such a kind of evolution before the intraoperative assessment.

The ossifications found above and within the substance of the tendon may be the result of a transformation of mesenchymal cells to bone-forming cells in response to the surgical excision of the calcium deposit and suturing of the tendon during the arthroscopic procedure.

Our preference for complete, meticulous excision of the mass might help avoid further recurrence of the ossifying mass. The long-term follow-up of the two cases described in this study showed no clinical or radiological recurrence of the deposits. Although the surgical approach may have been the trigger event inducing the chondrometaplasia, we have not enough data to support this speculative hypothesis, nor can we rule out that the ossification and cartilaginous metaplasia could be the natural evolution of the case. The surgical findings described in this study led us to consider with caution arthroscopic excision of calcium deposits and to be meticulous during the subacromial debridement of calcific foci to minimize the risk of recurrence. We do believe that the description of these two rare cases of OT will be useful to include this condition as a further complication of CT and also to consider the shoulder as an additional potential site of OT.

## References

[1] Painter CF (1907). Subdeltoid bursa. Bost Med Surg J.

[2] Codman EA (1909). Bursitis subacromialis, or periarthritis of the shoulder joint. Publications of the Mass Gen Hospital in Boston.

[3] Sandstrom C (1938). Peritendinitis calcarea: common disease of middle life: it’s diagnosis, pathology and treatment. Am J Roentgenol.

[4] DeSeze S, Welfing J (1970). Calcifying tendinitis. Rheumatologie.

[5] Bosworth B (1941). Calcium deposits in the shoulder and subacromial bursitis: a survey of 12,122 shoulders. JAMA.

[6] Uhthoff HK, Sarkar K, Maynard JA (1976). Calcifying tendinits: a new concept of its pathogenesis. Clin Orthop Relat Res.

[7] Ozaki J, Kugai A, Tomita Y, Tamai S (1992). Tear of an ossified rotator cuff of the shoulder. A case report.. Acta Orthop Scand.

[8] Erggelet C, Eggensperger G, Steinwachs M, Lahm A, Reichelt A (1999). Postoperative ossification of the shoulder. Incidence and clinical impact. Arch Orthop Trauma Surg.

[9] Ahmed SI, Burns TC, Landt C, Hayda R (2013). Heterotopic ossification in high-grade open fractures sustained in combat: risk factors and prevalence. J Orthop Trauma.

[10] Richards PJ, Braid JC, Carmont MR, Maffulli N (2008). Achilles tendon ossification: pathology, imaging and aetiology. Disabil Rehabil.

[11] Gallinet D, Dietsch E, Barbier-Brion B, Lerais JM, Obert L (2011). Suture anchor reinsertion of distal biceps rupture: clinical results and radiological assessment of tendon healing. Orthop Traumatol Surg Res.

[12] Krasny C, Enenkel M, Aigner N, Wlk M, Landsiedl F (2005). Ultrasound-guided needling combined with shock-wave therapy for the treatment of calcifying tendonitis of the shoulder. J Bone J Surg Br.

[13] Moretti B, Garofalo R, Genco S, Patella V, Mouhsine E (2005). Medium energy shock wave therapy in the treatment of rotator cuff calcifying tendonitis. Knee Surg Sports Traumatol Arthrosc.

[14] Daecke W, Kusnierczak D, Loew M (2002). Long-term effects of extracorporeal shockwave therapy in chronic calcific tendinitis of the shoulder. J Shoulder Elbow Surg.

[15] Sconfienza LM, Bandirali M, Serafini G, Lacelli F, Aliprandi A, Di Leo G (2012). Rotator cuff calcific tendinitis: does warm saline solution improve the short-term outcome of double-needle US-guided treatment?. Radiology.

[16] Porcellini G, Paladini P, Campi F, Paganelli M (2004). Arthroscopic treatment of calcifying tendinitis of the shoulder: clinical and ultrasonographic follow-up findings at two to five years. J Shoulder Elbow Surg.

[17] Seil R, Litzenburger H, Kohn D, Rupp S (2006). Arthroscopic treatment of chronically painful calcifying tendinitis of the supraspinatus tendon. Arthroscopy.

[18] Yoo JC, Park WH, Koh KH, Kim SM (2010). Arthroscopic treatment of chronic calcific tendinitis with complete removal and rotator cuff tendon repair. Knee Surg Sports Traumatol Arthrosc.

[19] Wittenberg RH, Rubenthaler F, Wolk T (2001). Surgical or conservative treatment for chronic rotator cuff calcifying tendinitis — a matched pair analysis of 100 patients. Arch Orthop Trauma Surg.

[20] Matsumoto I, Ito Y, Tomo H, Nakao Y, Takaoka K (2005). Case reports: ossified mass of the rotator cuff tendon in the subacromial bursa. Clin Orthop Relat Res.

[21] Degreef I, Debeer P (2006). Heterotopic ossification of the supraspinatus tendon after rotator cuff repair: a case report. Clin Rheumatol.

[22] Berg EE, Ciullo JV (1995). Heterotopic ossification after acromioplasty and distal clavicle resection. J Shoulder Elbow Surg.

[23] Sanders BS, Wilcox RB, Higgins LD (2010). Heterotopic ossification of the deltoid muscle after arthroscopic rotator cuff repair. Am J Orthop (Belle Mead NJ).

[24] Salingcarnboriboon R, Yoshitake H, Tsuji K, Obinata M, Amagasa T, Nifuji A, Noda M (2003). Establishment of tendon derived cell lines exhibiting pluripotent mesenchymal stem cell-like property. Exp Cell Res.

[25] Buring K (1975). On the origin of cells in heterotopic bone formation. Clin Orthop Relat Res.

[26] Craven PL, Urist MR (1971). Osteogenesis by radioisotope labelled cell populations in implants of bone matrix under influence of ionising radiation. Clin Orthop Relat Res.

[27] Neuwirth J, Fuhrmann RA, Veit A, Aurich M, Stonans I, Trommer T, Hortschansky P, Chubinskaya S, Mollenhauer JA (2006). Expression of bioactive bone morphogenetic proteins in the subacromial bursa of patients with chronic degeneration of the rotator cuff. Arthritis Res Ther.

[28] Lui PP, Chan LS, Cheuk YC, Lee YW, Chan KM (2009). Expression of bone morphogenetic protein-2 in the chondrogenic and ossifying sites of calcific tendinopathy and traumatic tendon injury rat models. J Orthop Surg Res.

[29] Mader R, Buskila D, Verlaan JJ, Atzeni F, Olivieri I, Pappone N, Di Girolamo C (2013). Developing new classification criteria for diffuse idiopathic skeletal hyperostosis: back to square one. Rheumatology (Oxford).

[30] Fuller DA, Mani US, Keenan MA (2013). Heterotopic ossification of the shoulder in patients with traumatic brain injury. J Shoulder Elbow Surg.

[31] Kircher J, Martinek V, Mittelmeier W (2007). Heterotopic ossification after minimally invasive rotator cuff repair. Arthroscopy.

